# Microhardness Variation with Indentation Depth for Body-Centered Cubic Steels Pertinent to Grain Size and Ferrite Content

**DOI:** 10.3390/ma17102371

**Published:** 2024-05-15

**Authors:** Anye Xu, Xuding Song, Min Ye, Yipin Wan, Chunguo Zhang

**Affiliations:** Key Laboratory of Road Construction Technology and Equipment, Ministry of Education, Chang’an University, Xi’an 710064, China; 2019225099@chd.edu.cn (A.X.); mingye@chd.edu.cn (M.Y.); wyipin@chd.edu.cn (Y.W.);

**Keywords:** indentation size effect, grain size, ferrite volume fraction, normal distribution

## Abstract

For a micro-indentation hardness test with non-destructivity, the Nix–Gao model is widely used to describe tested hardness or microhardness variation with an indentation depth induced by indentation size effect, in which tested hardness approaches the macrohardness when the indentation depth is large enough. Based on an analysis of hardness measurements on 10 body-centered cubic steels with diverse microstructure, this paper proposes an analytical relation between microhardness to macrohardness ratio and the indentation depth by explicitly linking characteristic indentation depth (a data-fitting parameter) to grain size and ferrite volume fraction using two different methods. In addition, the normal distribution theory is incorporated to consider the inevitable scatter of identical measurements resulting from material heterogeneity and machining/testing errors. Results show that the proposed model, with 96% reliability, can effectively predict microhardness variation with the indentation depth and its scatter.

## 1. Introduction

The hardness of a material as a mechanical constant should be independent of loading conditions. For micro- and nano-hardness measurements, however, the tested hardness value (*H*) varies with the indentation depth (*h*) due to indentation size effect (ISE) [1,2]. In general, *H* decreases with increasing *h*, and gradually tends to a steady-state value which is regarded as material constant or macrohardness (*H*_0_).

From the perspective of physics, the tested *H* value arises from the geometrically necessary dislocation (GND) and the statistically stored dislocation (SSD) of a material [3,4]. For the indentation test, strain gradient and corresponding GND increase with the decreasing indentation size [5,6]. That is why the tested *H* value continuously increases with the decreasing *h* value. For this reason, Nix and Gao developed strain gradient plasticity theory and proposed the below relation between *H*/*H*_0_ and *h* to describe the ISE phenomenon by considering the GND mechanism [7]:

(1) $$H=H_{0}\sqrt{1+\frac{h^{*}}{h}}$$

where the characteristic indentation depth (h^*^) can be obtained by data fitting.

Numerous studies were performed to study the factors related to ISE, e.g., microstructure of a material [8,9] and indenter geometry [10,11]. For instance, average grain size (*G*) and grain boundary significantly influence *H* value when *h* is comparable to *G* in size [8,12], and the length of GND and h* values are related to *G* [9]. However, the quantitative relationship between h* and *G* has not been determined. Chicot finds *H*_0_·$\sqrt{h}$^*^ is proportional to *μ*·$\sqrt{b}$ (*μ* is shear modulus, and *b* is Burgers vector), which indicates the h* value depends on the microstructure of a material [13]. Indenter geometry also has influences on the variation of the *H*–*h* curve, and a larger angle and bunting tip of an indenter result in slower *H* variation with *h* [14,15]. Based on the above, *μ* and *b* of a material and indenter angle are incorporated into the formula of h* to modify the Nix–Gao model [10]. While the limitation of the existing model needs to be fitted based on experimental data.

Body-centered cubic (BCC) steels with a matrix of α-Fe are widely used in industrial applications [16]. The material hardness of a BCC steel is also influenced by ferrite volume fraction (*V*_F_) [17]. An existing study finds that *V*_F_ influencing the h* value is inversely proportional to dislocation density [18], and SSD density is related to dislocation mean free path (*L*). For a material with *V*_F_ = 100%, *L* value approximates *G* in size [18,19]. That is, *G* and *V*_F_ are two significant factors related to h* value and ISE phenomenon.

**Figure 1:**
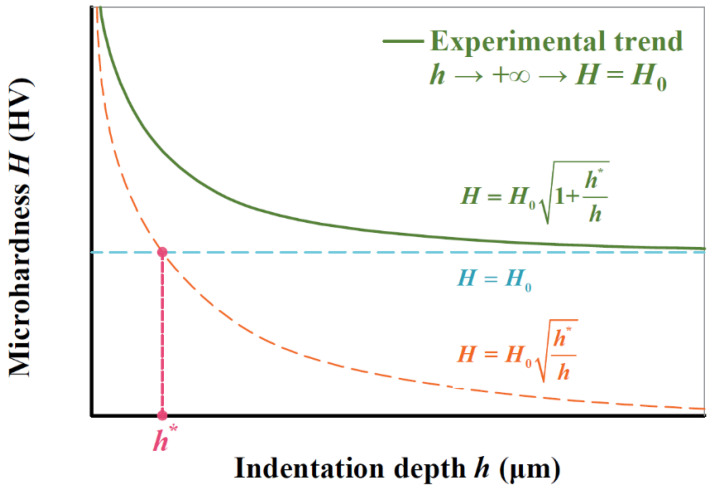
Nix–Gao model showing *H* variation with *h*.

In summary, h* in the Nix–Gao model is a data-fitting parameter which lacks a clear physical meaning. Based on the above discussions and in order to predict macrohardness *H*_0_, it is reasonable to link h* with *G* and *V*_F_ for BCC steels to determine an analytical description of the widely used Nix–Gao model. As illustrated in Figure 1, the intersection of two asymptotic curves is h*, indicating the translation from infinite hardness to *H*_0_. In the field of non-linear elastic fracture mechanics, the characteristic crack size (a*_ch_) indicates the transition from tensile strength-controlled to fracture toughness-controlled for the fracturing of brittle materials [20]. Interestingly, we find that a non-linear elastic fracture model and the Nix–Gao model are similar in form, and the characteristic parameter a^*^_ch_ is similar to h*. Our previous studies quantitatively link a^*^_ch_ with *G* for ceramics and obtain a quantitative relation of a^*^_ch_ = π·*G* through the analysis of experimental data [21]. Inspired by this, two methodologies: (i) theoretical derivation and (ii) the analysis of experimental data are adopted in this study to propose an analytical *H*_0_/*H*–*h* relation by explicitly linking h* to *G* and *V*_F_ based on the Nix–Gao model by analyzing Vickers hardness measurements on 10 BCC steels (*G* = 10.00–34.41 μm and *V*_F_ = 5–100%). Additionally, the normal distribution theory is incorporated into the model to consider the inevitable scatter in material properties resulting from material heterogeneity and machining/testing errors [22,23].

## 2. Experiments and Data

### 2.1. Measurements on Hardness, Grain Size and Ferrite Volume Fraction

Four BCC steels in Table 1: Q345B, 20 steel, and 45 steel from BaoWu Steel, Shanghai, China, and ER50-G from Golden Bridge Welding Materials, Tianjin are measured in this study. The microstructure of the steels is examined using an optical microscope after polishing and etching with 4% nital solution (4 mL HNO_3_ and 96 mL alcohol). Vickers hardness is measured by using a Wilson 402 MVD Vickers (Chang’an University, Xi’an, China) hardness tester with different loads (10 g, 25 g, 50 g, 100 g, 200 g, 300 g, 500 g, and 1 kg) with a dwell time of 20 s, and five measurements are performed under each load to get reliable results. According to ASTM E92 [24], the applying load (*P*) should be greater than 5 kg for *H*_0_ measurements. Thus, 10 kg is adopted to obtain *H*_0_ using a HV-10 Vickers hardness tester.

**Figure 2:**
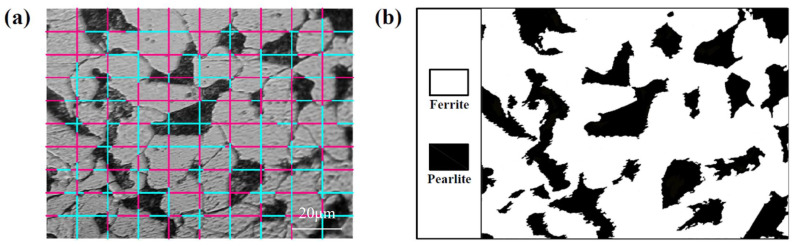
Illustrations of measuring (**a**) average grain size *G* and (**b**) ferrite volume fraction *V*_F_ for 20 steel.

Based on the microstructure image, *G* is measured using the intercept method according to ASTM E112 [25] (Figure 2a). *V*_F_ is obtained using the method shown in Figure 2b: an optical microstructure picture with two phases is converted to a black–white photo (white area indicates ferrite), and the proportion of the white area is *V*_F_ [19].

### 2.2. Experimental Data

**Figure 3:**
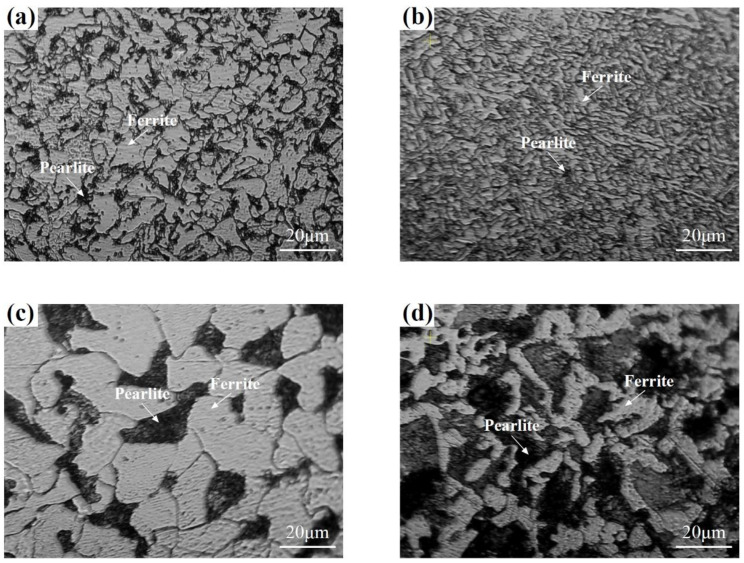

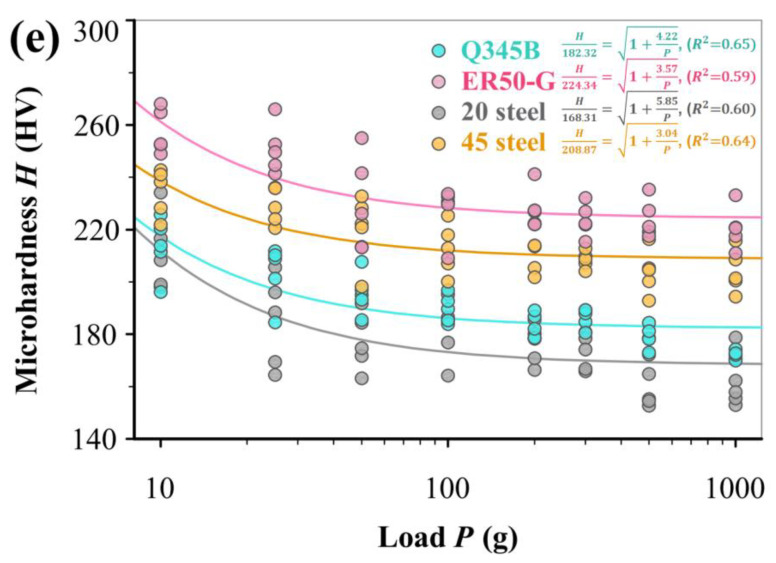
Microstructure of (**a**) Q345B, (**b**) ER50-G, (**c**) 20 steel and (**d**) 45 steel, and (**e**) Vickers microhardness variation with applying load.

Figure 3 shows the observed microstructure and *H* variation with *h* value for Q345B, ER50-G, 20 steel and 45 steel. The microstructure, *V*_F_, *G* and *H*_0_ of the four steels are listed in Table 2 together with those of other six BCC steels in the literature. It should be mentioned that only the maximum, minimum and mean values under each load are digitized for the hardness data (*h*, *H*) on the six BCC steels. The 10 BCC steels consist of up to two metallurgical structures (one of them is ferrite), in which Grade 91 10A and 10B steels consist mainly of martensite and a little ferrite [26,27], the high carbon steel with 0.71% C consists of spheroids cementite and ferrite [28], and the other seven steels consist of pearlite and ferrite.

## 3. Analytical *H*–*H*_0_ Relation by Linking h* to *G* and *V*_F_

To ensure the reliability, the analytical formula describing *H*/*H*_0_ variation with *h* is proposed based on two different methodologies (theoretical derivation and statistical analysis of experimental data) as shown in Section 3.1 and Section 3.2, respectively. In Section 3.3, the normal distribution analysis is incorporated in the formula to describe the inevitable scatter of measurements under identical conditions.

### 3.1. Theoretical Derivation

The ISE phenomenon during micro- and nano-hardness measurements can be formulated as Equation (1). Recent studies find that h* is related to microstructure, material properties and indenter geometry as follows [10]:

(2) $$h^{*}=\frac{81}{2}\frac{1}{f^{3}}\alpha^{2}b\tan^{2}\theta {\left(\frac{\mu }{H_{0}}\right)}^{2}$$

where the scaling factor *f* = *a*_PDZ_/*a* ≈ 1.9 (*a*_PDZ_ is the plastic zone size after indenting and *a* is indentation radius) for steels [32], indenter geometry parameter *θ* = 22° for Vickers indenter, and *α* ≈ 0.5, Burgers vector *b* ≈ 0.248 nm and shear modulus *μ* ≈ 80 GPa for BCC steels [12].

Yield strength *σ*_y_ of a material includes two parts [33] as follows:

(3) $$\sigma_{y}=\sigma_{\mathrm{GB}}+\sigma_{0}$$

where *σ*_GB_ is the contribution of grain boundaries to strength, and *σ*_0_ includes precipitation strengthening and solid solution strengthening. In the Hall–Petch relation, *σ*_GB_ is a function of average grain size *G* [34], as follows:

(4) $$\sigma_{\mathrm{GB}}=k_{y}G^{-0.5}$$

where the Hall–Petch coefficient *k*_y_ can be obtained by data fitting or the following formula [35]:

(5) $$k_{y}=\beta \mu \sqrt{b}$$

where *β* is a dimensionless data-fitting parameter. The parameter *k*_y_ ≈ 20 MPa$\sqrt{\mathrm{mm}}$ is for steels [36], thus *β* ≈ 0.5 is calculated based on Equation (5). In numerical terms, the contribution of grain boundaries to hardness (*H*_GB_) can be linked to *σ*_GB_ [34,36,37] as follows:

(6) $$H_{\mathrm{GB}}=3\sigma_{\mathrm{GB}}=3\beta \mu \sqrt{b}G^{-0.5}$$

It is easy to understand that the hardness of a material is pertinent to both metallography organization and grain size. For materials with ferrite, *V*_F_ has significant influence on the mechanical properties (e.g., hardness, yield strength, and plastic deformation). To consider both *G* and *V*_F_ influences, and inspired by the Hall–Patch relation, a concept of relative grain size (*G*_r_) is introduced in this study:

(7) $$G_{r}=G\cdot e^{\left(V_{F}-1\right)}$$

Thus, the following equation can be obtained by replacing *G* in Equation (6) by *G*_r_:

(8) $$H_{\mathrm{GB}}=3\beta \mu \sqrt{b}{\left(G_{r}\right)}^{-0.5}=3\beta \mu \sqrt{b}{\left(G\cdot e^{\left(V_{F}-1\right)}\right)}^{-0.5}$$

Similarly, Equation (2) can be rewritten as below by replacing *H*_0_ by *H*_GB_:

(9) $$h^{*}=\frac{81}{2}\frac{1}{f^{3}}\alpha^{2}b\;\tan^{2}\;\theta {\left(\frac{\mu }{3\beta \mu \sqrt{b}{\left(G\cdot e^{\left(V_{F}-1\right)}\right)}^{-0.5}}\right)}^{2}=\frac{81\alpha^{2}\;\tan^{2}\;\theta }{18f^{3}\beta^{2}}\cdot G\cdot e^{\left(V_{F}-1\right)}$$

For BCC steels with α-Fe as matrix, *f*, *θ*, and *α* in Equation (2) and *β* in Equation (5) are specific, thus h^*^ = 0.107 ≈ 0.1*G*·*e*^(*V*F−1)^. Then, Equation (1) can be rewritten as follows:

(10) $$H=H_{0}\sqrt{1+\frac{0.1G\cdot e^{\left(V_{F}-1\right)}}{h}}$$

### 3.2. Analysis of Experimental Data

Based on Taylor’s dislocation model and von Mises rule, tested hardness *H* resulted from GND and SSD, can be quantified as below [3]:

(11) $$H=3\sqrt{3}\alpha \mu b\sqrt{\rho_{G}+\rho_{S}}$$

where *ρ*_G_ is GND density and *ρ*_S_ is SSD density.

In Equation (11), *ρ*_G_ is a function of *h* [3].

(12) $$\rho_{G}=\frac{3}{2bh}\tan^{2}\theta$$

When *h* is infinite or large enough, *ρ*_G_ goes to zero, and *H* can be regarded as *H*_0_. That is, *H*_0_ is only governed by SSD, and Equation (11) can be rewritten as follows:

(13) $$H_{0}=3\sqrt{3}\alpha \mu b\sqrt{\rho_{S}}$$

In Equation (13), *ρ*_S_ can be linked to microstructure [18].

(14) $$\rho_{S}=\frac{1}{c^{*}bL}(1-e^{-3c^{*}\varepsilon^{*}})$$

where *L* is dislocation mean free path, and the representative strain ε* = 0.2∙tan*θ* = 0.08 for Vickers indentation tests [18,38], and c* can be obtained by data fitting. For a steel with *V*_F_ = 100%, *L* ≈ *G* [19], and the maximum value of *L* in a steel with *V*_F_ < 100% is not more than *G* value [39]. Based on the above discussion, it can be concluded that the *L* value is closely related to both *G* and *V*_F_ for BCC steels. Inspired by this, the following relation is assumed:

(15) $$L=G\cdot e^{\left(V_{F}-1\right)}$$

Combining Equations (11)–(15), the following equation is obtained:

(16) $$\frac{H}{H_{0}}=\sqrt{\frac{\rho_{S}+\rho_{G}}{\rho_{S}}}=\sqrt{1+\frac{\frac{3}{2bh}\tan^{2}\theta }{\frac{1-e^{-3c^{*}\varepsilon^{*}}}{c^{*}bG\cdot e^{\left(V_{F}-1\right)}}}}=\sqrt{1+\frac{C\cdot G\cdot e^{\left(V_{F}-1\right)}}{h}}$$

where *C* = 1.5c*tan^2^*θ*/(1 − *e*^−0.6c*tan*θ*^).

Comparing Equation (16) with Equation (1), h* = *CG*·*e*^(*V*F−1)^. The difference is that parameter h* in Equation (1) is a data-fitting parameter, and *CG*·*e*^(*V*F−1)^ in Equation (16) has an explicitly physical meaning if *C* is specific.

**Figure 4:**
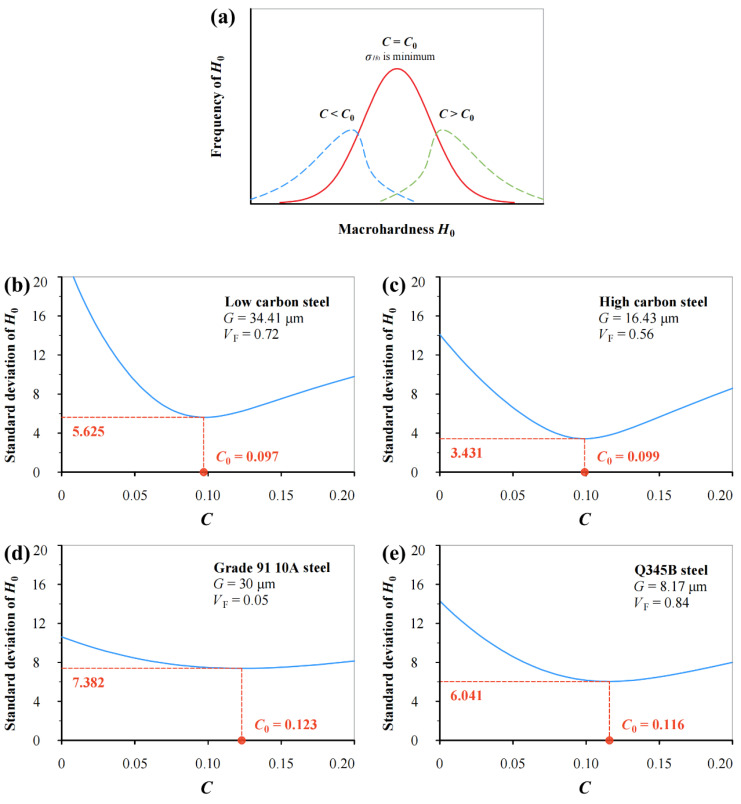
(**a**) Illustration of *H*_0_ variation with *C* value based on Equation (16) for (**b**) low carbon steel (0.19% C), (**c**) high carbon steel (0.71% C), (**d**) Grade 91 10A steel, and (**e**) Q345B steel.

As we all know, microhardness measurements on the same material under identical conditions inevitably fluctuate due to material heterogeneity and testing/machining errors. In Equation (16), *G* and *V*_F_ are specific for a material, and *H* and *h* can be directly obtained for an experiment. Thus, *H*_0_ value can be calculated using Equation (16) if *C* is a constant. It is easy to understand that *H*_0_ values should follow normal distribution for a group of measurements on a material. If the *C* value varies, the corresponding mean and stand deviation values will change for the same group of measurements. From a mathematic point of view, it is reasonable that a minimum of standard deviation (*σ_H_*_0_) exists with *C* variation when *C* becomes a critical value *C*_0_ as illustrated in Figure 4a.

To determine the specific value of *C*, measurements on low carbon steel (0.19% C), high carbon steel (0.71% C) and Grade 91 10A steel in the literature are analyzed together with measurements on Q345B steel in this study in Figure 4. The corresponding material parameters are listed in Table 2.

Clearly, the standard deviation of *H*_0_ (*σ_H_*_0_) varies continuously with the *C* value, and a critical value *C*_0_ exists, which corresponds to the minimum of *σ_H_*_0_. It can be seen that the *C*_0_ value varies from 0.097 to 0.123, and the average of the *C*_0_ values approximates 0.109.

**Figure 5:**
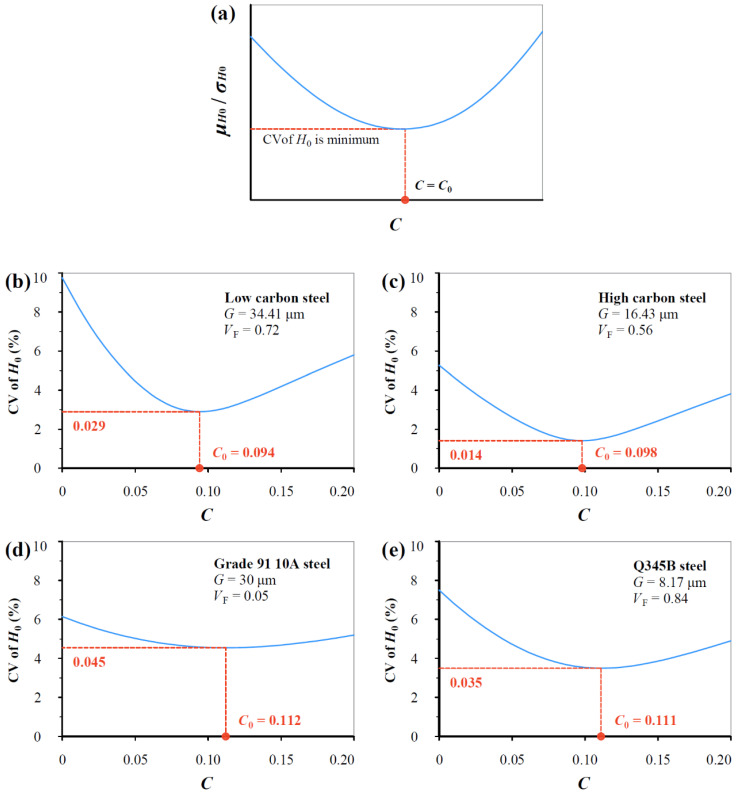
(**a**) Illustrating the CV variation with *C* value based on Equation (16) for (**b**) low carbon steel (0.19% C), (**c**) high carbon steel (0.71% C), (**d**) Grade 91 10A steel, and (**e**) Q345B steel.

Figure 5 shows coefficient of variation (CV = standard-deviation/mean) variation with the *C* value for the same data in Figure 4, which describes the relative scatter of data. Similarly, CV of *H*_0_ varies continuously with the *C* value, and a critical value *C*_0_ exists, which corresponds to the minimum of CV of *H*_0_. *C*_0_ value varies from 0.094 to 0.112 can be seen, and the average of the *C*_0_ values approximates 0.104.

**Figure 6:**
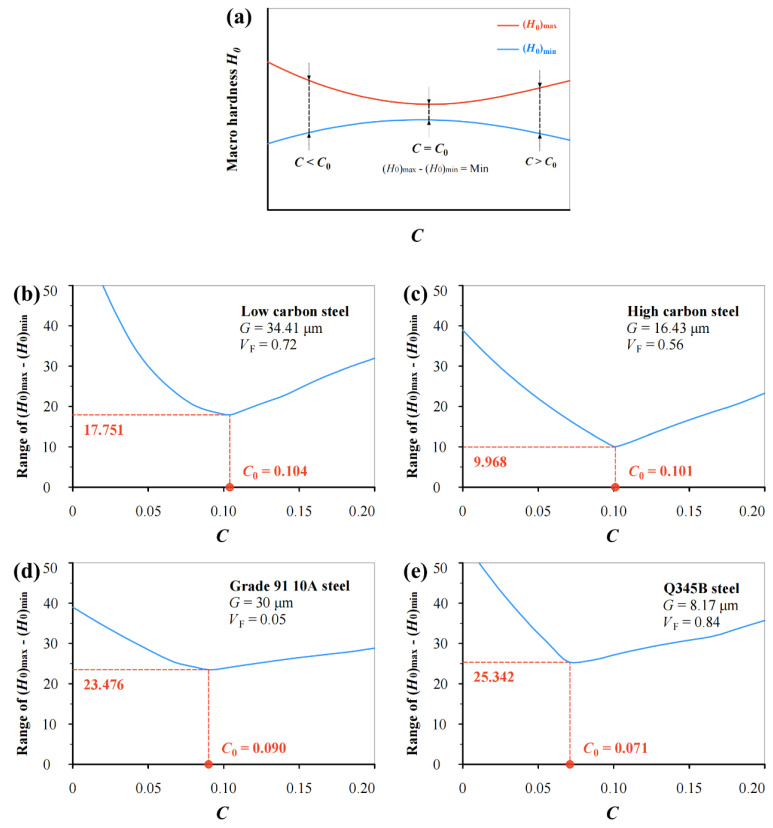
(**a**) Illustrating the variation in range of (*H*_0_)_max_–(*H*_0_)_min_ with *C* value based on Equation (16) for (**b**) low carbon steel (0.19% C), (**c**) high carbon steel (0.71% C), (**d**) Grade 91 10A steel, and (**e**) Q345B steel.

Figure 6 shows the variation in range of (*H*_0_)_max_–(*H*_0_)_min_ with the *C* value for the same data in Figure 4. Again, the minimum of (*H*_0_)_max_–(*H*_0_)_min_ occurs when *C* becomes a critical value *C*_0_. The variation in the *C*_0_ value from 0.071 to 0.104 can be seen, and the average of the *C*_0_ values approximates 0.092.

According to the above analyses, adopting three different mathematical statistical methods for the same measurements (four groups in total), it is found that the critical value *C*_0_ fluctuates around 0.1. For simplicity and consistency, it is reasonable to set *C*_0_ = 0.1 in this study. Thus, *H*/*H*_0_ variation is successfully described as linked to both *G* and *V*_F_, i.e., h* = 0.1*G*·*e*^(*V*F−1)^. That is, Equation (16) can be rewritten as Equation (10). It should be mentioned that the same equation can be obtained by using different methodologies in Section 3.1 and Section 3.2.

### 3.3. Normal Distribution Analysis for Inevitable Scatter of H/H_0_

In theory, the right side of Equation (10) is constant for a material under the same loading conditions, but the tested values of *H* on the left side inevitably fluctuate because of material heterogeneity and testing/matching errors. For this reason, a non-dimensional parameter *λ* is incorporated into Equation (10):

(17) $$\frac{H}{H_{0}}=\lambda \cdot \sqrt{1+\frac{0.1G\cdot e^{\left(V_{F}-1\right)}}{h}}=\lambda \cdot f(h,\;G,\;V_{F})$$

Obviously, the *H*/*H*_0_ ratio is fully determined by *G*, *V*_F_, and *h*. For a group of tested *H* values, corresponding *λ* values, calculated using Equation (17), can be analyzed using the normal distribution theory to obtain mean *μ_λ_* and standard deviation *σ_λ_*. Thus, Equation (17) can be re-reformulated to include both mean, and upper and lower bounds with 96% reliability:

(18) $$\frac{H}{H_{0}}=(\mu_{\lambda }\pm 2\sigma_{\lambda })\cdot \sqrt{1+\frac{0.1G\cdot e^{\left(V_{F}-1\right)}}{h}}=(\mu_{\lambda }\pm 2\sigma_{\lambda })\cdot f(h,\;G,\;V_{F})$$

**Figure 7:**
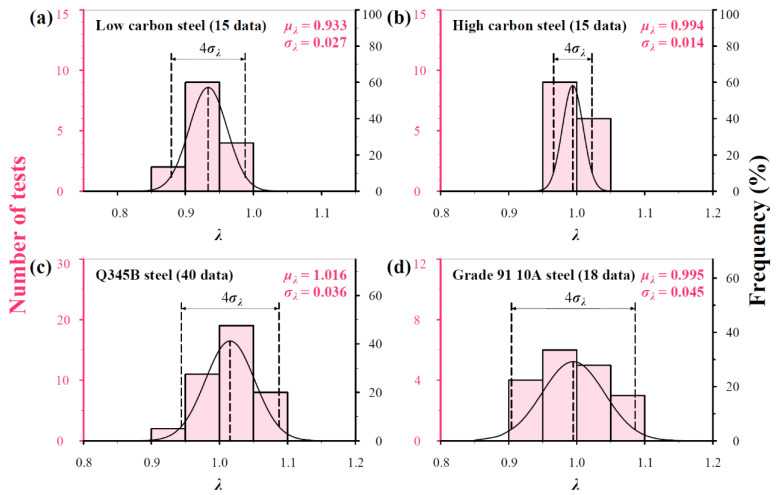
Histograms and frequencies of *λ* distributions from measurements on (**a**) low carbon steel (0.19% C), (**b**) high carbon steel (0.71% C), (**c**) Q345B steel, and (**d**) Grade 91 10A steel.

**Figure 8:**
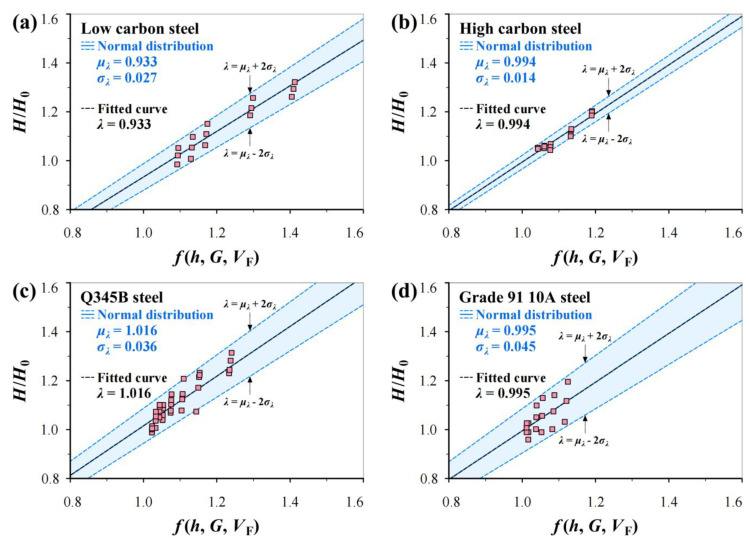
Predicted *H*/*H*_0_–*f*(*h*, *G*, *V*_F_) relations using Equation (18) in linearity using *μ_λ_* and *σ_λ_* values in Figure 7 together with fitted curves: (**a**) low carbon steel (0.19% C), (**b**) high carbon steel (0.71% C), (**c**) Q345B steel, and (**d**) Grade 91 10A steel.

To determine the values of *μ_λ_* and *σ_λ_*, the four groups of data in Figure 4 are reanalyzed in Figure 7 and Figure 8. It can be seen from Figure 7 that *μ_λ_* fluctuates from 0.933 to 1.106 and *σ_λ_* from 0.014 to 0.045. Figure 8 shows the predicted *H*/*H*_0_–*f*(*h*, *G*, *V*_F_) curves using Equation (18) in linearity using the *μ_λ_* and *σ_λ_* values in Figure 7 together with fitted curves for comparison, in which the *μ_λ_* ± 2*σ_λ_* values indicate the slopes of three predicted lines. It can be seen that the experimental points are almost covered by the predicted curves, in which the mean curves are nearly identical to fitted curves.

Although *μ_λ_* fluctuates from 0.933 to 1.106 for the four measurements, it should be 1.0 in theory. Considering the limitation of small sample data, the *σ_λ_* value should be no less than the maximum of 0.045 in Figure 7. For consistency, the rule of *μ_λ_* = 1.0 and *σ_λ_* = 0.05 is set in this study, thus Equation (18) can be rewritten as follows:

(19) $$\frac{H}{H_{0}}=(1.0\pm 2\times 0.05)\cdot \sqrt{1+\frac{0.1G\cdot e^{\left(V_{F}-1\right)}}{h}}=(1.0\pm 2\times 0.05)\cdot f(h,\;G,\;V_{F})$$

## 4. Verification

**Figure 9:**
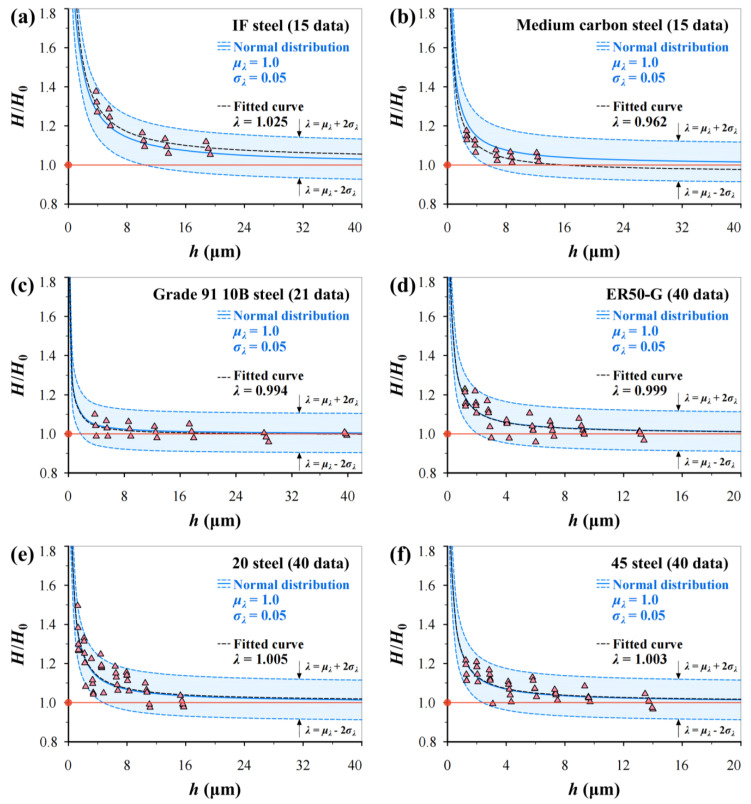
Predicted *H*/*H*_0_–*h* relations using Equation (19) together with fitted curves and experimental data: (**a**) IF steel, (**b**) medium carbon steel (0.32% C), (**c**) Grade 91 10B steel, (**d**) ER50-G, (**e**) 20 steel, and (**f**) 45 steel. The red line indicates a trend line.

To validate Equation (19), hardness measurements on six BCC steels in Table 2 are used: IF steel, medium carbon steel (0.32% C) and Grade 91 10B steel in the literature, and ER50-G, 20 steel and 45 steel in this study. Figure 9 shows the predicted *H*/*H*_0_–*h* curves using Equation (19) together with fitted curves and test data for comparison. It can be seen that the predicted curves with mean and upper and lower bounds cover most of the experimental data points.

**Figure 10:**
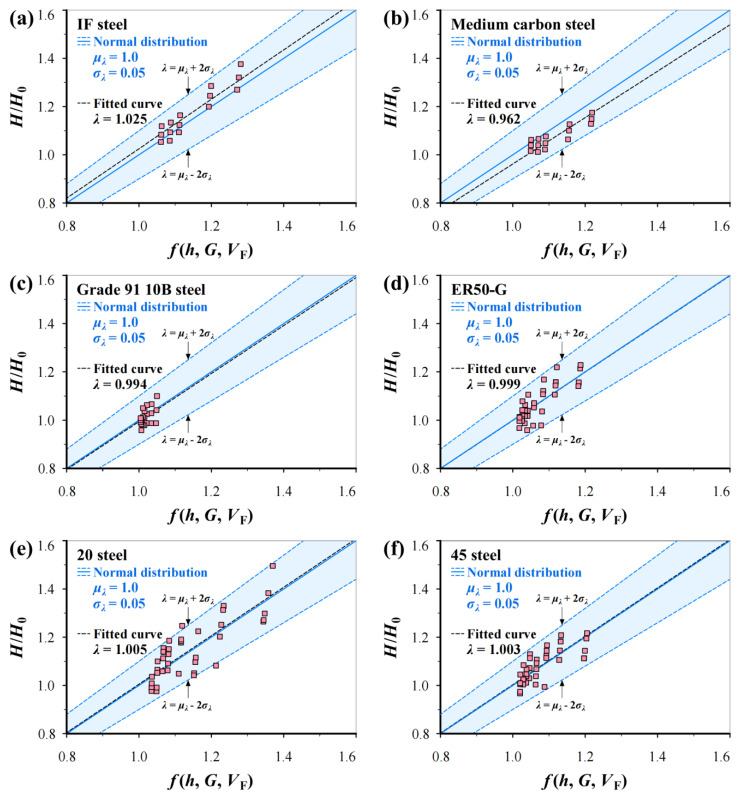
Predicted *H*/*H*_0_–*f*(*h*, *G*, *V*_F_) relations using Equation (19) in linearity together with fitted curves and experimental data: (**a**) IF steel, (**b**) medium carbon steel (0.32% C), (**c**) Grade 91 10B steel, (**d**) ER50-G, (**e**) 20 steel, and (**f**) 45 steel.

Figure 10 shows the predicted *H*/*H*_0_–*f*(*h*, *G*, *V*_F_) curves using Equation (19) in linearity without changing accuracy, in which 1.0 ± 2 × 0.05 indicates the slopes of three predicted lines. It can be seen that the predicted mean curves are very close to the corresponding fitted curves.

## 5. Discussion

For the micro-indentation hardness test, the ISE phenomenon cannot be ignored. The widely used Nix–Gao model can describe the microhardness variation; however, the empirical parameter h^*^ leads to a limitation of application for testing the material constant. Numerous studies show that tested *H* is not only related to the *h* value, but also related to the microstructure and phase content of a material. To investigate the hardness characteristics for BCC steels, which are widely used in industrial applications, h* is explicitly linked to *G* and *V*_F_ (h* = 0.1*G*·*e*^(*V*F−1)^) by two different methods to consider the grain size and phase content. The proposed model can be used to calculate the hardness of a material *H*_0_ based on tested data (*h*, *H*). Moreover, shallow indentation or low *h* value has no influence on the calculation accuracy of *H*_0_ but can significantly reduce the surface damage of materials or components. Thus, *H*_0_ can be easily obtained in microhardness tests with small load.

For BCC steels, larger *G* and higher *V*_F_ result in lower dislocation density leading to lower strength and higher plastic deformation capacity. Figure 9 shows that the *H*/*H*_0_–ratio with increasing *h* more easily approaches the steady state of one for Grade 91 10B steel than other steels. This is because Grade 91 10B has a mainly martensite structure, and *V*_F_ is lower than other materials, which results in a higher dislocation density. In fact, complex microstructural features such as secondary phases and carbide precipitation significantly affect test results. Thus, the influence of more factors on microhardness variation can be reflected by *H*. That is why only *G* and *V*_F_ are considered part of h^*^ of the Nix–Gao model and good robustness for different BCC steels can be found.

**Figure 11:**
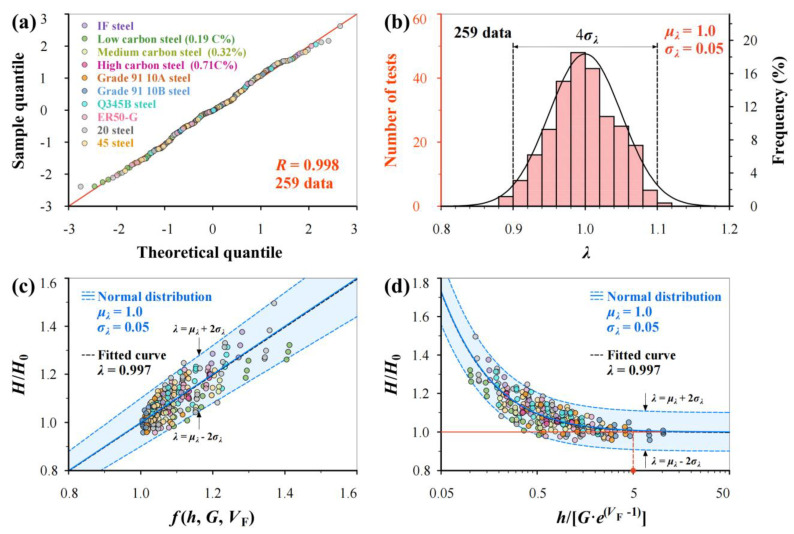
Integrated analysis of hardness measurements on 10 BCC steels. (**a**) q-q plot, (**b**) the histogram of *λ* values from total of 259 data and normal distribution prediction with *μ_λ_* = 1.0 and *σ_λ_* = 0.05, (**c**) predicted *H*/*H*_0_–*f*(*h*, *G*, *V*_F_) relations in linearity and (**d**) predicted *H*/*H*_0_–*h*/[*G*·*e*^(*V*F−1)^] curves using Equation (19) together with fitted curve and experimental data.

The scatter of hardness measurements under the same loading condition is inevitable due to the micro-heterogeneity of a material, and it increases with decreasing *h*. Therefore, normal distribution analysis is incorporated into the proposed model. To further validate Equation (19), Figure 11 shows the integrated analysis of measurements on 10 BCC steels. The q-q plot (Figure 11a) of *λ* values from all 259 data proves that the measurements on different materials still obeys normal distribution, as the correlation coefficient *R* = 0.998, and the unified normal distribution parameters (*μ_λ_* = 1.0 and *σ_λ_* = 0.05) are deemed acceptable by comparing the predicted curves with the histogram from measurements. Clearly, the prediction using Equation (19) agrees well with the experimental data, and the predicted curves with upper and lower bounds (96% reliability) cover most of the experimental points. Equation (19) can be flexibly used in linearity and non-linearity, as shown in Figure 11c,d. In addition, Figure 11d shows that the *H*/*H*_0_ ratio is less than 1.025 when *h*/[*G*∙*e*^(*V*F−1)^] > 5, in which the tested value *H* can be regarded as material hardness *H*_0_.

In addition, experimental data exhibiting a non-normal distribution can also be analyzed with Weibull distribution or lognormal distribution. For the application of other statistical models or distribution types, the non-dimensional parameters *μ_λ_* and *σ_λ_* can be modified by adding a skewness coefficient calculated from data.

## 6. Conclusions

This study investigates microhardness *H* variation in BCC steels with indentation depth *h* during micro-indentation hardness tests by analyzing Vickers microhardness measurements on 10 BCC steels with *G* from 10.00 μm to 34.41 μm and *V*_F_ from 5% to 100%. The main conclusions are as follows:

1. Based on the Nix–Gao model, an analytical relation between *H*/*H*_0_ and *h* is proposed, which can replace the fitting method commonly used. Through the proposed model, the hardness of a material *H*_0_ can be calculated based on tested data (*h*, *H*) under any loads.
2. The characteristic indentation depth h* indicates the translation from infinite hardness to macrohardness *H*_0_ in *H*-*h* curve. By two methods, h^*^ is explicitly linked to average grain size *G* and ferrite volume fraction *V*_F_ of BCC steels, i.e., h* = 0.1*G*∙*e*^(*V*F−1)^.
3. In micro-indentation hardness tests, when the indentation depth *h* is more than 5*G*∙*e*^(*V*F−1)^, the tested value *H* ≤ 1.025*H*_0_ which can be regarded as material hardness *H*_0_.
4. Normal distribution theory is incorporated successfully to quantify the inevitable scatter of hardness measurements resulting from the microstructure heterogeneity of a material and machining/testing errors. After considering scatter, this model includes both mean, and upper and lower bounds with 96% reliability, which ensue effective application for material testing and quality control.

## Figures and Tables

**Table 1:** Chemical compositions of materials tested in this study (in wt. %).

Materials	C	Si	Mn	P	S	Cu	Cr	Ni	Fe
Q345B	0.14	0.5	1.7	0.035	0.035	0.30	0.3	0.05	Balance *
ER50-G	0.07	0.9	1.5	0.012	0.011	0.5	0.02	0.02	Balance
20 steel	0.2	0.22	0.53	0.035	0.035	0.07	0.04	0.01	Balance
45 steel	0.45	0.17	0.5	0.035	0.035	0.25	0.25	0.25	Balance

* Balance: The proportion of elements other than those already listed.

**Table 2:** Material parameters of BCC steels used in this study [12,26,28,29,30,31].

Materials	Phase	*V*_F_	*G* (μm)	*H*_0_ (HV)
Q345B	F * + P *	84%	8.17	171.9
ER50-G	F + P	94%	5.19	218.2
20 steel	F + P	76%	14.14	156.6
45 steel	F + P	43%	10.01	199.5
IF steel [12]	F	100% [28]	24.29 [12]	94.5 [12]
Low carbon steel (0.19% C) [12]	F + P	72% [28]	34.41 [12]	206.7 [12]
Medium carbon steel (0.32% C) [12]	F + P	58% [28]	19.23 [12]	237.0 [12]
High carbon steel (0.71% C) [12]	F + SC *	56%	16.43 [12]	244.1 [12]
Grade 91 10A steel [29]	F + M *	5% [26]	30.00 [30]	164.7 [29]
Grade 91 10B steel [29]	F + M	5% [26]	10.00 [31]	240.0 [29]

* Ferrite (F), pearlite (P), spheroidal cementite (SC), and martensite (M).

## Data Availability

The raw data supporting the conclusions of this article will be made available by the authors on request.

## Nomenclature

*H*	Tested hardness or microhardness
*H*_0_	Material hardness or macrohardness
*h*	Indentation depth
*h* ^*^	Characteristic indentation depth as the translation from infinite hardness to *H*_0_
*G*	Average grain size
*V*_F_	Ferrite volume fraction
*b*	Burgers vector
*μ*	Shear modulus
*θ*	Indenter geometry parameter
*f*	Scaling factor
*σ*_y_	Yield strength
*σ*_GB_	The contribution of grain boundaries to strength
*σ*_0_	The contribution of strengthening other than grain boundaries strengthening.
*G*_r_	Relative grain size
*ρ*_G_	The density of geometrically necessary dislocation
*ρ*_S_	The density of statistically stored dislocation
*L*	Dislocation mean free path
*λ*	Non-dimensional parameter
*μ_λ_*	Mean of a group of *λ*
*σ_λ_*	Standard deviation of a group of *λ*
